# Draft genome sequence of *Methylobacterium aquaticum* LEGMi-203a, isolated from root nodules of *Pithecellobium hymenaeifolium*

**DOI:** 10.1128/mra.00754-25

**Published:** 2025-10-13

**Authors:** Valeria Castro-Camacho, Rubí Robles-Azor, Luciana Rodríguez-Burdock, Keilor Rojas-Jimenez, Bradd Mendoza-Guido

**Affiliations:** 1 Escuela de Biología, Universidad de Costa Rica 27915, , San José, Costa Rica; 2 Instituto de Investigaciones en Salud, Universidad de Costa Rica 27915, San José, Costa Rica; University of Southern California, Los Angeles, California, USA

**Keywords:** symbiosis, nitrogen fixation, Costa Rica

## Abstract

We report the draft genome of *Methylobacterium aquaticum* LEGMi-203a, a root nodule isolated from *Pithecellobium hymenaeifolium*. Genomic analysis supports its classification as *M. aquaticum,* and annotated nitrogen fixation and nodulation genes underscore its possible functional capabilities as a symbiont in tropical plants.

## ANNOUNCEMENT

The genus *Methylobacterium* includes species known for plant growth promotion and facilitation of symbiosis within root nodules (1). *M. aquaticum* has shown the ability to utilize methanol, a by-product of plant metabolism, as a carbon source, emphasizing its relevance in symbiotic interactions (2). Genomic analysis has revealed the evolutionary and functional diversity of *Methylobacterium*, highlighting their plant-associated traits (3).

We obtained the isolate in August 2021 from root nodules of the native legume *P. hymenaeifolium* (Michiguiste) in Costa Rica (10°00′50″N 84°07′53″W). We sampled four plant nodules, surface-sterilized, macerated, and serially diluted them (10⁻¹–10⁻²) in Peptone Yeast Extract (PY) broth. Aliquots (50 µL) were plated on PY agar supplemented with cycloheximide (40 mg/L) and incubated at 28°C for up to 3 weeks. Colonies were repeatedly streaked on PY agar and preserved in PY broth with 20% glycerol at −80°C. A loopful of the bacterial strain was then collected from the solid culture, resuspended in 250 µL PBS, and genomic DNA was extracted using the DNEasy PowerSoil Kit (Qiagen, USA) according to the manufacturer’s protocol.

Libraries were prepared using the Illumina DNA Prep kit, following the manufacturer’s protocol. Genomic DNA was randomly sheared, end repaired, A-tailed, and ligated with Illumina adapters. Adapter-ligated fragments were PCR-amplified, size-selected, purified, and assessed using Qubit, real-time PCR, and Bioanalyzer. Quantified libraries were pooled, and paired-end whole-genome sequencing (150 bp reads) was performed on the Illumina NovaSeq 6000 platform at Novogene Co. (CA, USA).

Sequencing produced 13,714,316 raw reads that were assessed for quality using fastp v0.20.1 (4), with average quality threshold of 30 and assembled using SPAdes v3.15.4 (5) with k-mer values of 21, 33, 55, and 77. Functional annotation was performed using PGAP v6.10 (6). Default parameters were used for all software except where otherwise noted.

**Fig 1:**
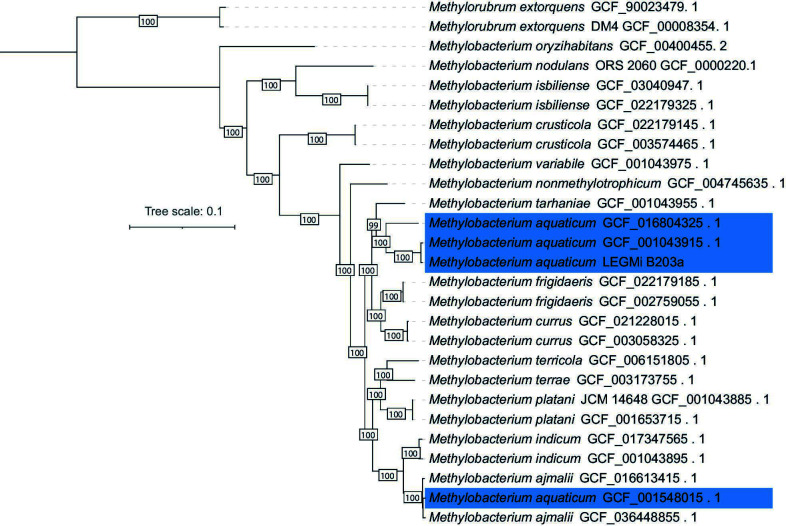
Phylogenomic tree of 27 genomes closely related to *M. aquaticum*, based on clade C1. The tree was constructed with FastTree v2.1.11 (10) using amino acid sequences and 891 single-copy core genes. Visualization and edition were conducted in iTOL v6 (13). Bootstrap values are shown in the nodes.

We performed phylogenomic analysis employing two strains from each species in *Methylobacterium* clade C1 (7) as reference, and three *M. aquaticum* genomes available in the NCBI database (Fig. 1) to confirm taxonomy. We used an in-house pipeline that selects all the single-copy core genes (SCCGs) in the pangenome of the data set applying a MCL inflation value of 2 (8). The SCCGs were aligned with MAFFT v7.397 (9) and used to generate a phylogenomic tree with FastTree v2.1.10 employing the General Time Reversible model and 1,000 bootstrap resamplings (10). Average nucleotide identity (ANI) was evaluated with pyANI (11) and digital DNA-DNA hybridization (dDDH) with the TYGS web server (12).

The *M. aquaticum* LEGMi-203a strain genome is 7,778,826 bp long with 7,913 putative coding sequences. The assembly resulted in 151 contigs, an N50 value of 150114, L50 of 17, a GC content of 69.8%, and an average coverage of 237.511. PGAP identified genes linked to nitrogen fixation and nodulation, including *nifU*-like domains, the transcriptional regulator *FixJ*, and the N-related dehydratase *MaoC*. Our isolate appeared related to the *M. aquaticum* type strain (DSM16371^t^) (ANI 98.9%, dDDH 83.3%) (Fig. 1).

## Data Availability

This whole-genome sequence project has been deposited in GenBank under the accession no. ASM5040874v1. The raw sequencing reads were deposited under the accession number SRX26808260.
